# Supplementary material for: Role of solvent-anion charge transfer in oxidative degradation of battery electrolytes
Source: Nat Commun. 2019 Jul 26;10:3360. doi: 10.1038/s41467-019-11317-3 (PMC6659707; doi:10.1038/s41467-019-11317-3)
Supplement: Supplementary file 1 — Supplementary Information [file 41467_2019_11317_MOESM1_ESM.pdf]

Supplementary Information

Role of Solvent-Anion Charge Transfer in Oxidative Degradation of Battery Electrolytes

E. R. Fadel et al.

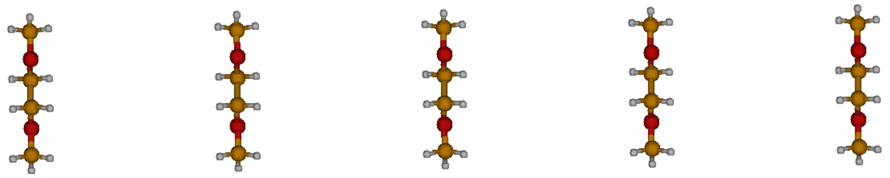

Supplementary Figure 1: DME_5 system used to study IP of DME. Five identical DME molecules are placed 10 Å apart. Different DFT methods provide different descriptions of the ionization process.

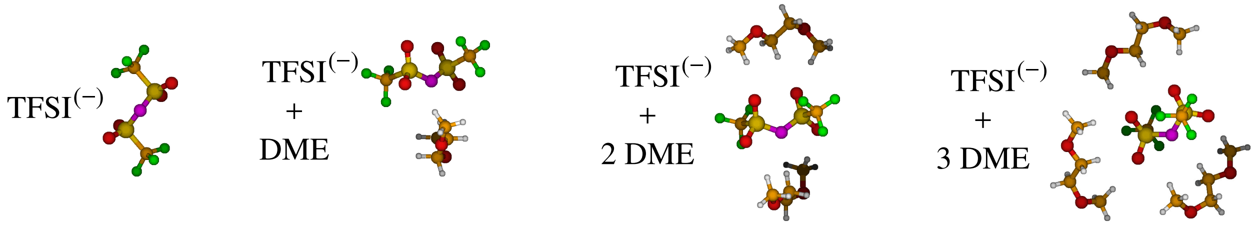

Supplementary Figure 2: Geometries used to investigate ionization of TFSI^{-} surrounded by DME solvating molecules. Although intermolecular distances depend on the method used, all optimized geometries are qualitatively similar.

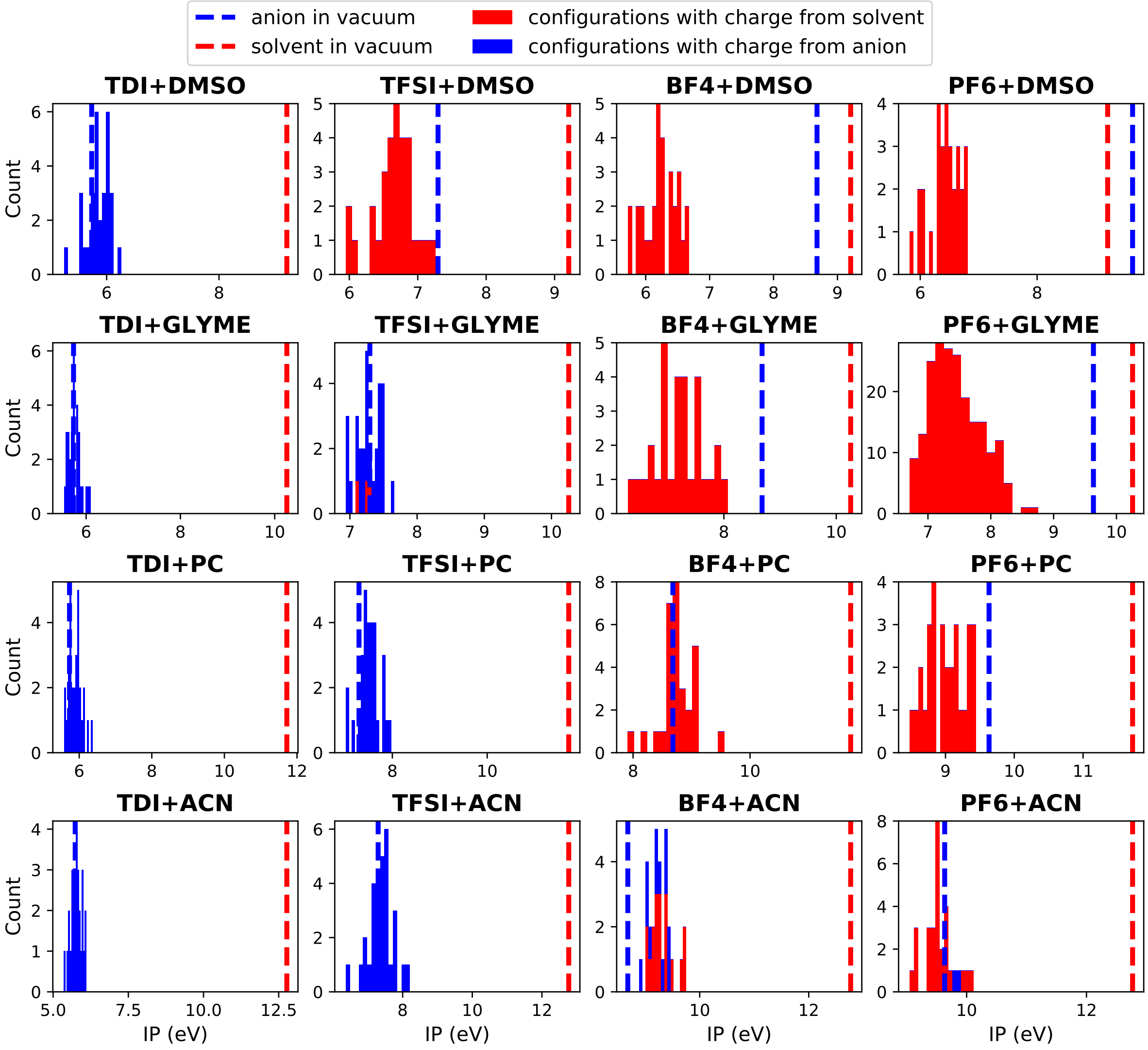

Supplementary Figure 3: Histogram plots of anion-solvent couple vertical IP. The vertical IP for the anion in vacuum and solvent in vacuum are also shown. This study is done over 4x4 electrolytes (anions: TDI^- , $TFSI^-$, BF_4^- , PF_6^- ; solvents: DMSO, GLYME, PC, ACN).

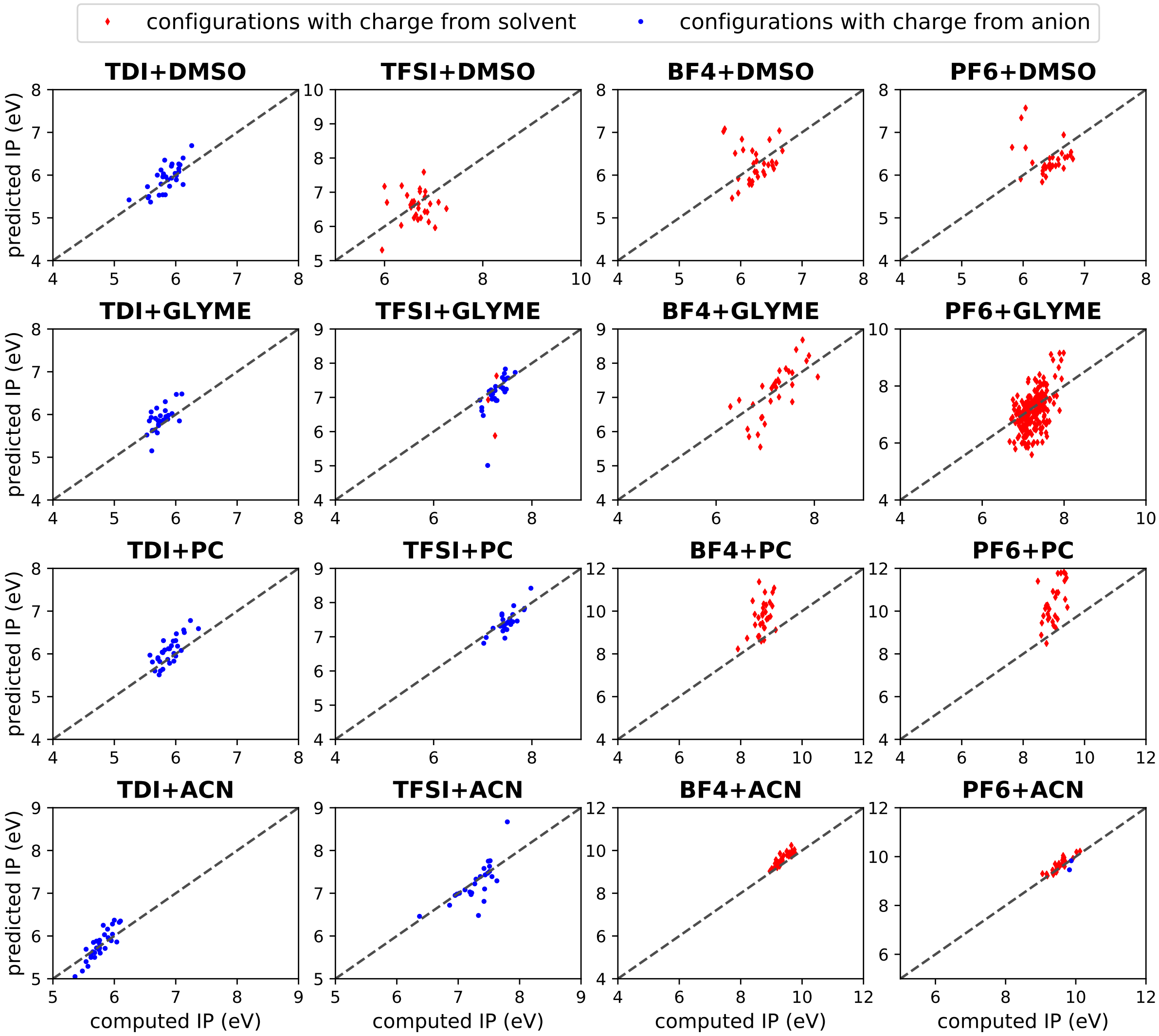

Supplementary Figure 4: Plot of the predicted IP from the simple ionization model presented in this work against the computed IP ($IP_{\Delta SCF}$) for all 16 couples considered in this work.

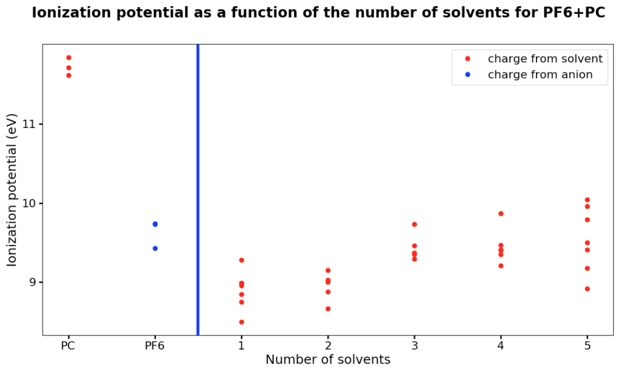

Supplementary Figure 5: IP of different configurations of PF_6^- solvated by one to five PC molecules. The color represents which species yielded the charge, and the isolated IP for the molecule are represented to the left.

Supplementary Table 1: Ionization Potential (in eV) for DME and DME_5, and charge distribution for DME_5. Each DME is represented by a box ( ) with darkness proportional to the amount of charge lost upon ionization. Only LC-BLYP, HF, and M06-HF provide the correct qualitative description.

Method	IP(DME)	IP(DME_5)	Charge Distribution (%)				
PBE	8.75	6.77	23	18	18	18	23
PBE0	9.34	8.06	23	18	18	18	23
B3LYP	9.30	7.88	23	18	18	18	23
B3LYP-D3	9.30	7.88	23	18	18	18	23
M06-2X	9.92	9.36	24	17	17	17	24
CAM-B3LYP	9.73	9.16	23	18	18	18	23
LC-BLYP	10.13	10.11	0	0	100	0	0
M06-HF	10.24	10.20	0	0	100	0	0
HF	8.82	8.80	0	0	100	0	0
MP2	10.27	–					
CCSD(T)[a]	9.88	–					

[a] CCSD geometry

Supplementary Table 2: Ionization Potential (in eV) for $TFSI^-$ and $(TFSI^-)_5$, and charge distribution for $(TFSI^-)_5$. Each $TFSI^-$ is represented by a box ( ) with darkness proportional to the amount of charge lost upon ionization. Only methods with long range HF exchange provide a qualitatively correct description.

Method	IP($TFSI^-$)	IP[$(TFSI^-)_5$]	Charge Distribution (%)				
PBE	5.82	3.60	20	20	20	20	20
PBE0	6.37	4.85	20	20	20	20	20
B3LYP	6.85	4.70	20	20	20	20	20
B3LYP-D3	6.85	4.71	20	20	20	20	20
M06-2X	7.26	6.22	19	20	21	20	19
CAM-B3LYP	6.72	6.08	19	21	21	21	19
LC-BLYP	7.17	7.08	0	0	100	0	0
M06-HF	7.67	7.66	0	0	100	0	0
HF	6.30	6.17	0	0	100	0	0
MP2	7.02	–					
CCSD(T)[a]	6.75	–					

[a] CCSD geometry

Supplementary Table 3: Ionization Potential (eV) and charge distribution for systems comprising TFSI$^-$ and a varying number of DMEs, represented as and , respectively, in vacuum. The shade of gray of each box is proportional to the amount of charge removed from the corresponding molecule, also reported as percentage value inside each box.

Method	DME: IP	DME	TFSI$^-$: IP	TFSI$^-$	DME/TFSI$^-$: IP	DME	TFSI$^-$	DME/TFSI$^-$/DME: IP	DME (top)	TFSI$^-$	DME (bottom)	DME DME/TFSI$^-$/DME: IP	DME (top left)	DME (top right)	TFSI$^-$	DME (bottom)
PBE	8.75	100	5.82	100	5.26	54	46	5.00	42	17	41	4.99	38	35	6	21
PBE0	9.34	100	6.86	100	6.08	56	44	6.03	49	4	47	6.07	36	35	5	22
B3LYP	9.30	100	6.85	100	6.05	54	46	5.90	43	15	42	5.91	35	34	5	22
B3LYP-D3	9.30	100	6.85	100	5.95	62	38	5.81	48	6	45	5.80	35	33	2	30
M06-2X	9.92	100	7.26	100	6.96	71	29	6.82	50	7	43	6.85	34	35	2	30
CAM-B3LYP	9.73	100	6.72	100	6.65	62	38	6.77	53	7	40	6.86	41	34	7	17
LC-BLYP	10.13	100	7.17	100	6.91	95	5	7.13	96	3	1	7.23	93	0	2	4
M06-HF	10.24	100	7.93	100	7.07	96	4	7.25	96	3	1	7.27	95	2	0	3
HF	8.82	100	6.30	100	5.95	99	1	6.16	99	1	0	6.45	99	0	1	0

Supplementary Table 4: Ionization Potential (eV) and charge distribution for systems comprising $TFSI^-$ and a varying number of DMEs, represented as [box] and [box], respectively, with implicit solvation in diethylether.

Method	DME IP	DME	$TFSI^-$ IP	$TFSI^-$	DME/$TFSI^-$ IP	DME	$TFSI^-$	DME/$TFSI^-$/DME IP	DME (top)	$TFSI^-$	DME (bottom)	DME DME/$TFSI^-$/DME IP	DME (top left)	DME (top right)	$TFSI^-$	DME (bottom)
PBE	7.20	100	7.18	100	6.32	68	32	6.03	48	10	42	6.14	55	35	10	1
PBE0	7.80	100	8.17	100	7.18	76	24	7.36	50	2	48	6.95	35	38	5	22
B3LYP	7.75	100	7.72	100	7.06	81	19	6.95	55	2	42	6.90	33	43	2	23
B3LYP-D3	7.76	100	7.73	100	6.97	83	17	6.97	51	5	44	6.74	34	31	1	33
M06-2X	8.31	100	8.33	100	7.50	97	3	7.55	95	4	1	7.56	97	1	1	1
CAM-B3LYP	8.17	100	8.07	100	7.43	98	2	7.49	97	3	0	7.50	96	0	3	1
LC-BLYP	8.37	100	8.52	100	7.59	100	0	7.63	99	0	0	7.70	95	0	2	2
M06-HF	8.50	100	8.50	100	7.71	99	1	7.74	97	3	0	7.73	97	2	0	1
HF	7.03	100	7.57	100	6.47	100	0	6.42	100	0	0	6.51	100	0	0	0

Supplementary Table 5: Ionization Potential (eV) and charge distribution for systems comprising PF_6^- and a varying number of DMEs, represented as and , respectively, with implicit solvation in diethylether.

Method	DME: IP	DME: charge	PF_6^-: IP	PF_6^-: charge	DME–PF_6^-: IP	DME	PF_6^-	DME–PF_6^-–DME: IP	DME (top)	PF_6^-	DME (bottom)	4 DME–PF_6^-: IP	DME (top left)	DME (top right)	PF_6^-	DME (bottom left)	DME (bottom right)
PBE	7.20	100	8.69	100	6.24	99	1	5.67	50	0	50	5.47	25	26	0	24	25
PBE0	7.80	100	9.80	100	6.75	100	0	6.59	50	0	50	6.55	26	27	0	24	23
B3LYP	7.75	100	9.71	100	6.78	100	0	6.51	50	0	50	6.45	26	24	0	25	25
B3LYP-D3	7.76	100	9.87	100	6.61	99	1	6.38	50	1	50	6.32	26	27	-2	25	24
M06-2X	8.31	100	10.71	100	7.03	99	1	7.06	99	0	1	7.20	1	5	-3	93	5
CAM-B3LYP	8.17	100	10.24	100	7.03	100	0	7.07	99	0	0	7.07	2	1	0	97	0
LC-BLYP	8.37	100	10.95	100	7.36	100	0	7.39	100	0	0	7.54	1	4	-3	94	3
M06-HF	8.50	100	11.70	100	7.53	100	0	7.53	99	0	1	7.67	1	2	-2	98	2
HF	7.03	100	10.10	100	6.44	100	0	6.41	0	0	100	6.50	99	0	0	0	0

Supplementary Table 6: RMSD (Å) between the coordinates obtained with various DFT methods and MP2 or CCSD for dimer systems.

	MP2				CCSD
	$TFSI^-$ -DME[a]	$TFSI^-$ -DME[b]	PF_6^- -DME[b]	Average	PF_6^- -DME[b]
PBE	0.34	0.40	0.24	0.33	0.24
PBE0	0.32	0.25	0.24	0.27	0.24
B3LYP	0.56	0.50	0.42	0.49	0.42
B3LYP-D3	0.12	0.11	0.24	0.16	0.23
M06-2X	1.42	1.47	0.13	1.00	0.14
CAM-B3LYP	0.42	0.36	0.15	0.31	0.14
LC-BLYP	0.11	0.25	0.09	0.15	0.09
M06-HF	0.18	0.11	0.26	0.18	0.26
HF	0.54	0.67	0.53	0.58	0.52

[a] in vacuum; [b] in diethylether.

Supplementary Table 7: Values of $\delta_\bullet$ for all pairs with BF_4^- anion. $\delta_\bullet$ is defined as the IP shift that is the difference of the pair IP (in implicit solvation) and the IP of the lone solvent (in implicit solvation) in the case where the solvent is oxidized (column 1). This is averaged over 5 different configurations picked close to the IP distribution peak for the pair (and in the case of ACN, only configurations leading to solvent oxidation).

Couples	$\delta_\bullet$
BF_4^- +DMSO	0.1
BF_4^- +DME	0.64
BF_4^- +PC	0.12
BF_4^- +ACN	0.16

Supplementary Table 8: DLPNO-CCSD(T) vertical IP for the 4 solvents and 4 anions, averaged over 25 configurations. Columns 1 and 5 show the different solvent and anion species respectively. Column 2 shows experimental values for solvents IP from photo-emission spectroscopy.[1] Columns 3 and 6 are M06-HF IP values for solvents and anions respectively. Columns 4 and 7 are DLPNO-CCSD(T) IP values for solvents and anions respectively.

Solvent	Exp. IP	M06-HF IP	CCSD(T) IP	Anion	M06-HF IP	CCSD(T) IP
DMSO	9.0-9.1	9.0	8.8	TDI^-	5.7	5.0
DME	9.8-9.9	10.6	9.9	$TFSI^-$	7.3	6.7
PC	-	12.0	11.0	BF_4^-	9.4	8.6
ACN	12.2-12.5	12.4	12.2	PF_6^-	10.2	8.9

Supplementary Table 9: DLPNO-CCSD(T) vertical $IP_{\Delta SCF}$ for the couples (PC,TDI^-), (PC,$TFSI^-$), (PC,BF_4^-) and (PC,PF_6^-) (rows 3, 4, 5 and 6). Columns 2, 3 and 4 show the isolated PC, isolated anion and anion-solvent pair IP from DLPNO-CCSD(T) calculations for the different couples. Columns 5, 6 and 7 show the same for M06-HF in comparison.

Method	CCSD(T) IP			M06-HF IP		
Couples	PC	Anion	Pair	PC	Anion	Pair
PC+TDI^-	11	5.0	4.9	11.7	5.6	5.9
PC+$TFSI^-$	11	6.7	6.6	11.7	7.2	7.5
PC+BF_4^-	11	8.6	8.2	11.7	8.8	8.7
PC+PF_6^-	11	8.9	8.3	11.7	9.7	9.0

Supplementary Note 1 – Charge Delocalization Study DFT functional comparison

Specific details about the methods used in this section are presented in the Supplementary Methods section. First, we consider the ionization of DME in vacuum. For each form of DFT, we optimized the geometry of one DME molecule, and computed the vertical IP. We then placed five copies of the same DME molecule 10 Å apart, as shown in Supplementary Figure 1. This system is referred to as DME_5. The IPs obtained for DME_5 are compared to those for DME in Supplementary Table 1, where we also report the charge distribution in the ionized state.

Within a single Slater determinant approximation for the wavefunction, the electron is expected to be removed from one molecule (the one at the center) with an IP almost identical to that for DME. A more complete description including resonance of five Slater determinants would delocalize the charge on all five molecules, but the IP would still correspond to the removal of one electron from one of the molecules, hence it would not change significantly. By contrast, removing a fraction of an electron from each of the DMEs yields a smaller IP which depends on the number of DMEs considered. We find that only methods including full long range HF exchange (LC-BLYP, M06-HF, and HF) correctly remove the electron from a single DME molecule. For these, the IP is almost independent from the number of molecules considered. The slight decrease computed for DME_5 is likely due to polarization of the nearby molecules.

Next, we consider ionization of the anion $TFSI^-$ in vacuum. In order to reduce coulomb interactions between the charged molecules, we placed five anions 500 Å apart. For this system, called $(TFSI^-)_5$, the electrostatic interaction energy is under 0.2 eV. The IPs for single $TFSI^-$ and for $(TFSI^-)_5$ are reported in Supplementary Table 2.

Again, to obtain a qualitatively correct description, i.e., ionization of one anion, it is essential to include long range HF exchange: of the methods considered, only LC-BLYP, M06-HF, and HF correctly remove one electron from the central anion. The other methods

remove approximately one fifth of an electron from each of the five anions, which is incorrect.

Next we investigate ionization considering one $TFSI^-$ anion surrounded by DME solvating molecules. The optimized geometries for $TFSI^-$ and between zero and three DMEs depend on the computational method used, but they are all qualitatively similar, and are reported schematically in Supplementary Figure 2.

The corresponding ionization potentials and charge distribution are in Supplementary Table 3.

In agreement with literature reports,[2,3] methods without full HF exchange misrepresent the ionized state, predicting charges delocalized on more than one molecule. When ionization occurs, LC-BLYP, HF, and M06-HF remove the electron from one of the DMEs. All other functionals delocalize the charge, removing a fraction of an electron from all molecules. When only one DME molecule is included, a significant fraction of the charge comes from $TFSI^-$. As more DMEs are added, $TFSI^-$ looses less negative charge upon ionization.

We now add implicit solvation, which favors charge separation, and find that it enhances this effect. We report in Supplementary Table 4 the ionization potentials and charge distributions derived using diethylether as implicit solvent.

The general trend is qualitatively similar to that computed in vacuum, except for M06-2X and CAM-B3LYP. For these functional, implicit solvation leads to charge and spin localization on one of the DMEs. Not surprisingly, implicit solvation raises the IP for $TFSI^-$ and lowers that for DME. This results in similar IPs for the two species even neglecting their interactions (second column in Supplementary Table 4. Hence, the fraction of charge coming from $TFSI^-$ is generally less than for the corresponding cases with no implicit solvent. For instance, at the PBE0 level, $TFSI^-$ with one DME looses 24% and 44% of an electron from $TFSI^-$ with and without implicit solvation, respectively.

For functionals with less than 50% of HF exchange, the charge removed from DME tends to be delocalized on all DME molecules available.

In summary, all functionals predict ionization to occur from the solvent within the first

solvation shell of the anion, but only functionals with long range HF exchange correctly localize the charge in the ionized state. An immediate consequence is that the IP is expected to be practically independent from the anion used.

To test this conclusion, we performed similar IP computations for the anion PF_6^- surrounded by 0, 1, 2, and 4 DME molecules. The results are summarized in Supplementary Table 5.

Since IP(PF_6^-) is larger than IP(TFSI$^-$), practically all of the charge is removed from DME, even using functionals with no HF exchange, that tend to delocalize the charge. For systems with more than one DME, however, we obtain the same behavior seen with TFSI$^-$, i.e., functionals with low HF exchange delocalize the charge on all DMEs available, and functionals with high HF exchange localize the ionization from one DME. Again, we find that M06-2X and CAM-B3LYP localize the charge when implicit solvation is added and delocalize it in vacuum (non reported in Supplementary Table 5.

Using LC-BLYP or M06-HF as reference, we compute IP(PF_6^- /DME) $\approx$ $7.5 - 7.7\ eV$, quite similar to IP(TFSI$^-$ /DME) $\approx$ $7.7\ eV$.

Since IPs depend on the electrostatic stabilization between TFSI$^-$ and DME$^+$, as is highlighted in the main paper, it is important to describe correctly the geometry for these systems. So, to assess the different methods, we compare the DFT geometries for dimers TFSI$^-$ -DME and PF_6^- -DME with MP2 geometries. The root mean squared geometric difference (RMSD)[4,5] is reported in Supplementary Table 6, where average values refer to the arithmetic mean for each row.

Of the methods considered, B3LYP-D3, LC-BLYP, M06-HF and, to some extent, PBE0, predict geometries similar to MP2. Considering that B3LYP-D3 and PBE0 yield a wrong description of the ionized state, we conclude that only LC-BLYP and M06-HF are appropriate to study these systems. Both these methods predict an IP for the electrolyte near an anion (TFSI$^-$ or PF_6^-) of approximately $7.7\ eV$, with the electron being removed from the DME molecule nearest to the anion.

The likelihood for this ionization to occur and the chemical consequences in terms of

electrolyte degradation will be the subject of an upcoming separate publication.

To summarize this comparative study, we tested nine different forms of DFT and their ability to describe ionization of an anion in solution, as relevant for the electrochemical failure of lithium-metal rechargeable batteries. We find that only functionals including some form of self interaction correction at long range provide the correct physical description of the ionization process, i.e., removal of one electron from one molecule. Functionals with 100% of HF exchange at long range (HF, M06-HF, LC-BLYP) yield the correct answer even in vacuum; those with less than 50% of HF exchange (PBE, PBE0, B3LYP, B3LYP-D3) appear to unduly delocalize the charge over several molecules, leading to a dependence of the IP on system size. M06-2X, with 54%, and CAM-B3LYP, with 65% of HF exchange at long range, provide the correct description when implicit solvation is used but fail in vacuum. Among the functionals providing the correct qualitative description, LC-BLYP and M06-HF appear to yield the best coordinating geometries, compared to ab-initio MP2 or CCSD.

Supplementary Note 2 – Charge Transfer Model

Here we detail the mathematics of the charge transfer model. The ionization potential of a species A in vacuum can be decomposed into a quantum energy part (that we note $E_q(A)$) and a classical electrostatic part ($E_e(A)$), that is the contribution to the energy from generating an electric field in vacuum. Since A is a molecule, electrostatic fields in the form of higher order dipole moments will exist even in the neutral case.

$$\mathrm{IP}\left(A^0\right) = E_{total}\left(A^+\right) - E_{total}\left(A^0\right) = \overbrace{E_q\left(A^+\right) - E_q\left(A^0\right)}^{quantum} + \overbrace{E_e\left(A^+\right) - E_e\left(A^0\right)}^{electrostatic}$$

(Supplementary Equation 1)

When looking at a combined system of two molecules A and S where A represents an anion and S a neutral solvent, the IP can be decomposed also into a quantum and classical

electrostatic part. We make the assumption that A and S are decoupled (if they are sufficiently far apart which we expect to be the case for a solvated anion). We also assume that A is the negatively charged species initially (this is just a convention on which species holds the charge), and that in the final state, the lowest energy state may be the ionization of the anion or the solvent. Therefore the correct final energy is the minimum of the two cases. With these conventions and assumptions, the IP of the combined system is a minimum of two values:

$$\begin{aligned}
\mathrm{IP}\left([A^- S^0]\right) &= \min \begin{cases} \overbrace{E_q\left([A^0 S^0]\right) - E_q\left([A^- S^0]\right)}^{\text{quantum}} + \overbrace{E_e\left([A^0 S^0]\right) - E_e\left([A^- S^0]\right)}^{\text{electrostatic}} \\ \underbrace{E_q\left([A^- S^+]\right) - E_q\left([A^- S^0]\right)}_{\text{quantum}} + \underbrace{E_e\left([A^- S^+]\right) - E_e\left([A^- S^0]\right)}_{\text{electrostatic}} \end{cases} \\
&= \min \begin{cases} E_q(A^0) - E_q(A^-) + E_e([A^0 S^0]) - E_e([A^- S^0]), & \text{if the anion is ionized} \\ E_q(S^+) - E_q(S^0) + E_e([A^- S^+]) - E_e([A^- S^0]), & \text{if the solvent is ionized.} \end{cases}
\end{aligned}$$

(Supplementary Equation 2)

Introducing the electrostatic energies for the isolated molecules, one can find a more interesting expression for the IP of the couple as a function of the isolated IP:

$$\mathrm{IP}\left([A^- S^0]\right) = \min \begin{cases} \mathrm{IP}(A^-) + \overbrace{E_e\left([A^0 S^0]\right) - E_e\left(A^0\right) - E_e\left(S^0\right)}^{E^{\mathrm{bind}}\left([A^0 S^0]\right)} \overbrace{- E_e\left([A^- S^0]\right) + E_e\left(A^-\right) + E_e\left(S^0\right)}^{-E^{\mathrm{bind}}\left([A^- S^0]\right)} \\ \mathrm{IP}(S^0) + \underbrace{E_e\left([A^- S^+]\right) - E_e\left(A^-\right) - E_e\left(S^+\right)}_{E^{\mathrm{bind}}([A^- S^+])} \underbrace{- E_e\left([A^- S^0]\right) + E_e\left(A^-\right) + E_e\left(S^0\right)}_{-E^{\mathrm{bind}}([A^- S^0])} \end{cases}$$

(Supplementary Equation 3)

We now focus on the charge transfer model generalization in solution, In the study done in vacuum, we have highlighted an interesting property of the IP of the couple compared to that of the isolated species. In order to show this behavior, it was necessary to focus only on the couple, by representing it explicitly, and not using implicit solvation elsewhere.

Therefore, the impact of electrostatics in the interaction of anion and solvent is different in this study (i.e. in vacuum) than in solution. Here, we discuss how the results found here are affected by solvation. It has been shown in this study that whenever more solvent are explicitly added to the system, there is still one and only one species that is fully ionized upon removal of charge (whether that is the anion or one of the solvent). Supplementary Figure 5 shows the IP for the (PF_6^- ,PC) couple for different number of explicit solvents (up to five), and illustrates this observation.

In this discussion, at first, we keep all solvation energy terms, and we note:

$$E_e\left(A_{\bullet}^{-}\right) = E_e\left(A^{-}\right) + \Delta\left(A^{-}\right)$$
$$E_e\left([A^{-}S^{0}]_{\bullet}\right) = E_e\left([A^{-}S^{0}]\right) + \Delta\left([A^{-}S^{0}]\right)$$
$$E_e\left([A^{-}S^{+}]_{\bullet}\right) = E_e\left([A^{-}S^{+}]\right) + \Delta\left([A^{-}S^{+}]\right)$$
$$E_e\left(S_{\bullet}^{+}\right) = E_e\left(S^{+}\right) + \Delta\left(S^{+}\right)$$

where the subscript $\bullet$ denotes the solvated species (anion or solvent), the solvation energy of molecule A is $\Delta\left(A\right)$.

Recalling the formula derived in the previous section, relating the IP of the couple to the IPs of the isolated species, and introducing the solvation energy, it is possible to rewrite all the terms involved:

$$\mathrm{IP}\left([A^-S^0]_\bullet\right) = \min \begin{cases} E\left([A^0S^0]\right) - E\left([A^-S^0]\right) + \Delta\left([A^0S^0]\right) - \Delta\left([A^-S^0]\right) \\ E\left([A^-S^+]\right) - E\left([A^-S^0]\right) + \Delta\left([A^-S^+]\right) - \Delta\left([A^-S^0]\right) \end{cases}$$

$$\mathrm{IP}\left(A^-_\bullet\right) = I\mathrm{IP}\left(A^-\right) - \Delta\left(A^-\right) + \Delta\left(A^0\right)$$

$$\mathrm{IP}\left(S^0_\bullet\right) = \mathrm{IP}\left(S^0\right) + \Delta\left(S^+\right) - \Delta\left(S^0\right)$$

$$\begin{aligned} E^{\mathrm{bind}}\left([A^0S^0]\right) &= E_e\left([A^0S^0]\right) - E_e\left(A^0\right) - E_e\left(S^0\right) \\ &= E_e\left([A^0S^0]_\bullet\right) - \Delta\left([A^0S^0]\right) - E_e\left(A^0_\bullet\right) + \Delta\left(A^0\right) - E_e\left(S^0_\bullet\right) + \Delta\left(S^0\right) \\ &= E^{\mathrm{bind}}\left([A^0S^0]_\bullet\right) - \Delta\left([A^0S^0]\right) + \Delta\left(A^0\right) + \Delta\left(S^0\right) \end{aligned}$$

$$\begin{aligned} E^{\mathrm{bind}}\left([A^-S^0]\right) &= E_e\left([A^-S^0]\right) - E_e\left(A^-\right) - E_e\left(S^0\right) \\ &= E_e\left([A^-S^0]_\bullet\right) - \Delta\left([A^-S^0]\right) - E_e\left(A^-_\bullet\right) + \Delta\left(A^-\right) - E_e\left(S^0_\bullet\right) + \Delta\left(S^0\right) \\ &= E^{\mathrm{bind}}\left([A^-S^0]_\bullet\right) - \Delta\left([A^-S^0]\right) + \Delta\left(A^-\right) + \Delta\left(S^0\right) \end{aligned}$$

$$\begin{aligned} E^{\mathrm{bind}}\left([A^-S^+]\right) &= E_e\left([A^-S^+]\right) - E_e\left(A^-\right) - E_e\left(S^+\right) \\ &= E_e\left([A^-S^+]_\bullet\right) - \Delta\left([A^-S^+]\right) - E_e\left(A^-_\bullet\right) + \Delta\left(A^-\right) - E_e\left(S^+_\bullet\right) + \Delta\left(S^+\right) \\ &= E^{\mathrm{bind}}\left([A^-S^+]_\bullet\right) - \Delta\left([A^-S^+]\right) + \Delta\left(A^-\right) + \Delta\left(S^+\right) \end{aligned}$$

And thus the IP of the fully solvated anion-solvent couple may be expressed in a very similar expression to the vacuum case, involving the IP of the solvated anion and solvent, as

well as the dipole energy of the solvated couple:

$$
\begin{aligned}
\mathrm{IP}\left([A^-S^0]_\bullet\right) &= \min\begin{cases} E\left([A^0S^0]\right) - E\left([A^-S^0]\right) + \Delta\left([A^0S^0]\right) - \Delta\left([A^-S^0]\right)) \\ E\left([A^-S^+]\right) - E\left([A^-S^0]\right) + \Delta\left([A^-S^+]\right) - \Delta\left([A^-S^0]\right) \end{cases} \\
&= \min\begin{cases} \mathrm{IP}\left(A^-\right) + E^{\mathrm{bind}}\left([A^0S^0]\right) - E^{\mathrm{bind}}\left([A^-S^0]\right) + \Delta\left([A^0S^0]\right) - \Delta\left([A^-S^0]\right) \\ \mathrm{IP}\left(S^0\right) + E^{\mathrm{bind}}\left([A^-S^+]\right) - E^{\mathrm{bind}}\left([A^-S^0]\right) + \Delta\left([A^-S^+]\right) - \Delta\left([A^-S^0]\right) \end{cases} \\
&= \min\begin{cases} \mathrm{IP}\left(A^-\right) + \overbrace{E^{\mathrm{bind}}\left([A^0S^0]\right) + \Delta\left([A^0S^0]\right)} - E^{\mathrm{bind}}\left([A^-S^0]\right) - \Delta\left([A^-S^0]\right) \\ \mathrm{IP}\left(S^0\right) + \underbrace{E^{\mathrm{bind}}\left([A^-S^+]\right) + \Delta\left([A^-S^+]\right)} - E^{\mathrm{bind}}\left([A^-S^0]\right) - \Delta\left([A^-S^0]\right) \end{cases} \\
&= \min\begin{cases} \mathrm{IP}\left(A^-\right) + E^{\mathrm{bind}}\left([A^0S^0]_\bullet\right) + \Delta\left(A^0\right) + \Delta\left(S^0\right) - E^{\mathrm{bind}}\left([A^-S^0]\right) - \Delta\left([A^-S^0]\right) \\ \mathrm{IP}\left(S^0\right) + E^{\mathrm{bind}}\left([A^-S^+]_\bullet\right) + \Delta\left(A^-\right) + \Delta\left(S^+\right) - E^{\mathrm{bind}}\left([A^-S^0]\right) - \Delta\left([A^-S^0]\right) \end{cases} \\
&= \min\begin{cases} \mathrm{IP}\left(A^-\right) + \Delta\left(A^0\right) + E^{\mathrm{bind}}\left([A^0S^0]_\bullet\right) - \overbrace{\left(E^{\mathrm{bind}}\left([A^-S^0]\right) + \Delta\left([A^-S^0]\right) - \Delta\left(S^0\right)\right)} \\ \mathrm{IP}\left(S^0\right) + \Delta\left(S^+\right) + E^{\mathrm{bind}}\left([A^-S^+]_\bullet\right) - \underbrace{\left(E^{\mathrm{bind}}\left([A^-S^0]\right) + \Delta\left([A^-S^0]\right) - \Delta\left(A^-\right)\right)} \end{cases} \\
&= \min\begin{cases} \mathrm{IP}\left(A^-\right) + \Delta\left(A^0\right) + E^{\mathrm{bind}}\left([A^0S^0]_\bullet\right) - \left(E^{\mathrm{bind}}\left([A^-S^0]_\bullet\right) + \Delta\left(A^-\right)\right) \\ \mathrm{IP}\left(S^0\right) + \Delta\left(S^+\right) + E^{\mathrm{bind}}\left([A^-S^+]_\bullet\right) - \left(E^{\mathrm{bind}}\left([A^-S^0]_\bullet\right) + \Delta\left(S^0\right)\right) \end{cases} \\
&= \min\begin{cases} \mathrm{IP}\left(A^-\right) + \Delta\left(A^0\right) - \Delta\left(A^-\right) + E^{\mathrm{bind}}\left([A^0S^0]_\bullet\right) - E^{\mathrm{bind}}\left([A^-S^0]_\bullet\right) \\ \mathrm{IP}\left(S^0\right) + \Delta\left(S^+\right) - \Delta\left(S^0\right) + E^{\mathrm{bind}}\left([A^-S^+]_\bullet\right) - E^{\mathrm{bind}}\left([A^-S^0]_\bullet\right) \end{cases} \\
&= \min\begin{cases} \mathrm{IP}\left(A^-_\bullet\right) + E^{\mathrm{bind}}\left([A^0S^0]_\bullet\right) - E^{\mathrm{bind}}\left([A^-S^0]_\bullet\right) \\ \mathrm{IP}\left(S^0_\bullet\right) + E^{\mathrm{bind}}\left([A^-S^+]_\bullet\right) - E^{\mathrm{bind}}\left([A^-S^0]_\bullet\right) \end{cases}
\end{aligned}
$$

Such that:

$$\mathrm{IP}\left([A^-S^0]_\bullet\right) = \min \begin{cases} \mathrm{IP}\left(A^-_\bullet\right) + E^{\mathrm{bind}}\left([A^0S^0]_\bullet\right) - E^{\mathrm{bind}}\left([A^-S^0]_\bullet\right) \\ \mathrm{IP}\left(S^0_\bullet\right) + E^{\mathrm{bind}}\left([A^-S^+]_\bullet\right) - E^{\mathrm{bind}}\left([A^-S^0]_\bullet\right) \end{cases}$$

(Supplementary Equation 4)

Consider now the case where the solvent is oxidized. In the main article, the decrease in IP is noted δ. Let us note $\delta_\bullet$ the decrease in IP when considering the couple in solvation. From our previous equations, we can determine the change in δ:

$$\delta = \mathrm{IP}\left(S^0\right) - \mathrm{IP}\left([A^-S^0]\right) = E^{\mathrm{bind}}\left([A^-S^0]\right) - E^{\mathrm{bind}}\left([A^-S^+]\right)$$

$$\delta_\bullet = \mathrm{IP}\left(S^0_\bullet\right) - \mathrm{IP}\left([A^-S^0]_\bullet\right) = E^{\mathrm{bind}}\left([A^-S^0]_\bullet\right) - E^{\mathrm{bind}}\left([A^-S^+]_\bullet\right)$$

We note that these two quantities are positive. Indeed, the classical electrostatic binding energy of a dipole charge-transfer complex $[A^-S^+]$ is expected to be larger in absolute value than that of the unoxidized $[A^-S^0]$ pair. This is verified in the computations highlighted in the article, where we find that for all cases where the solvent is oxidized, the IP of the couple is lower than that of the isolated solvent. As presented in the article, across all couples studied here, the difference in IP is 2.8 eV on average in vacuum, and always positive also in the solvated cases. For all couples with the BF_4^- anion, we compute the value of $\delta_\bullet$ with 5 configurations of anion-solvent pairs and using implicit solvation to approximate the solvation of this pair, using the method described in the methods section of the manuscript to obtain the compute the change on the vertical IP. The static and high-frequency dielectric constants (ϵ_0,ϵ_∞) for the solvents are taken to be (46.8,4.16) for DMSO, for (4.24,2.16) DME, (65.5,4.14) for PC and (35.7,4.0) for ACN. We find that the solvent is still the oxidized species, just like in the vacuum case, and the value of the difference $\delta_\bullet$ between the IP of the solvent and that of the solvent-anion pair is indeed lower but still significant in the PCM-solvated calculations. The results are presented in the Supplementary Table 7.

Supplementary Note 3 – DLPNO-CCSD(T) validation of the findings

DLPNO-CCSD(T) calculations were performed to validate our computational approach and our findings. These calculations were performed as described in the methods section of the main paper. Firstly, we show that vertical IP calculations using DLPNO-CCSD(T) calculations agree very well with experimental IP values for solvents. Furthermore, these results allow us to benchmark M06-HF for the chemistries studied. We find that our DFT calculations perform well for the chemistries studied. These results are summarized in Supplementary Table 8.

Thus, it is possible to accurately describe the IP of isolated species using a more costly computational method. Furthermore, when using this very reliable computational method, the trends presented in this work are exactly the same. To prove this, we performed the same type of calculations as in the main paper, focusing on the anion-solvent pairs with PC solvent. We compute the average $IP_{\Delta SCF}$ over 20 configurations (using the same configurations as for the M06-HF analysis) and compare with our DFT calculations. The results are presented in Supplementary Table 9.

Similarly to the M06-HF DFT results, the anion is oxidized in the (TDI^- , PC) and ($TFSI^-$, PC) cases, with the pair IP close to the anion IP (since the difference between isolated anion and solvent IP is greater than δ). At the same time, the solvent is oxidized in the (BF_4^- , PC) and (PF_6^- , PC) cases with an average pair IP equal to the solvent IP minus $\delta = 2.8eV$. Thus, higher-order DLPNO-CCSD(T) computations show excellent agreement with the DFT conclusions, and validate our approach. The range of IP pair values due to geometry effects is similarly within about 1eV. This indicates that configuration geometry effects are large compared to the energy difference between the M06-HF and CCSD(T) approximations.

Supplementary Methods

The computational details for this work are highlighted in the main paper. This supplementary Method section highlights specific details about the calculations presented in Supplementary Tables 1 to 6. We performed computations both in vacuum and in diethylether, with a dielectric constant $\epsilon = 4.24$, similar to that of PEO, treated as implicit solvent.[6]

We used the following DFT methods, each comprising the amount of HF exchange indicated in parenthesis. Since it includes long range SIC, HF exchange is important for a correct description of charge localization in the ionized state.[2,3]

PBE[7,8] (0%), **B3LYP**[9] (20%), **B3LYP-D3**,[10] with Grimme dispersion correction (20%), **CAM-B3LYP**[11] (19% at short range, 65% at long range), **PBE0**[12] (25%), **M06-2X**[13] (54%), **LC-BLYP**[14,15] (0% at short range, 100% at long range), **M06-HF**[16,17] (100%), **HF** (100%.) Also, on selected small systems we used MP2,[18] CCSD,[19] and CCSD(T)[20] for validation purposes.

Supplementary References

(1) Linstrom, P. J. & Mallard, W. G. The NIST Chemistry WebBook: A chemical data resource on the internet. *J. Chem. Eng. Data* **46**, 1059–1063 (2001).

(2) Whittleton, S. R., Sosa Vazquez, X. A., Isborn, C. M. & Johnson, E. R. Density-functional errors in ionization potential with increasing system size. *J. Chem. Phys.* **142**, 184106 (2015).

(3) Sosa Vazquez, X. A. & Isborn, C. M. Size-dependent error of the density functional theory ionization potential in vacuum and solution. *J. Chem. Phys.* **143**, 244105 (2015).

(4) Kromann, J. C. & Bratholm, A. Calculate RMSD for two XYZ structures. *http://github.com/charnley/rmsd* (2017).

(5) Kabsch, W. A solution for the best rotation to relate two sets of vectors. *Acta Crystallogr. A* **32**, 922–923 (1976).

(6) Tomasi, J., Mennucci, B. & Cammi, R. Quantum mechanical continuum solvation models. *Chem. Rev.* **105**, 2999–3094 (2005).

(7) Perdew, J. P., Burke, K. & Ernzerhof, M. Generalized gradient approximation made simple. *Phys. Rev. Lett.* **77**, 3865 (1996).

(8) Perdew, J. P., Burke, K. & Ernzerhof, M. Generalized Gradient Approximation Made Simple [Phys. Rev. Lett. 77, 3865 (1996)]. *Phys. Rev. Lett.* **78**, 1396 (1997).

(9) Becke, A. D. Becke's three parameter hybrid method using the LYP correlation functional. *J. Chem. Phys.* **98**, 5648–5652 (1993).

(10) Grimme, S., Antony, J., Ehrlich, S. & Krieg, H. A consistent and accurate ab initio parametrization of density functional dispersion correction (DFT-D) for the 94 elements H-Pu. *J. Chem. Phys.* **132**, 154104 (2010).

(11) Yanai, T., Tew, D. P. & Handy, N. C. A new hybrid exchange–correlation functional using the Coulomb-attenuating method (CAM-B3LYP). *Chem. Phys. Lett.* **393**, 51–57 (2004).

(12) Adamo, C., & Barone, V. Toward reliable density functional methods without adjustable parameters: The PBE0 model. *J. Chem. Phys.* **110**, 6158–6170 (1999).

(13) Zhao, Y. & Truhlar, D. G. The m06 suite of density functionals for main group thermochemistry, thermochemical kinetics, noncovalent interactions, excited states, and transition elements: two new functionals and systematic testing of four m06-class functionals and 12 other functionals. *Theor. Chem. Acc.* **120**, 215–241 (2008).

(14) Iikura, H., Tsuneda, T., Yanai, T. & Hirao, K. A long-range correction scheme for

generalized-gradient-approximation exchange functionals. *J. Chem. Phys.* **115**, 3540–3544 (2001).

(15) Becke, A. D. Density-functional exchange-energy approximation with correct asymptotic behavior. *Phys. Rev. A* **38**, 3098 (1988).

(16) Zhao, Y. & Truhlar, D. G. Comparative dft study of van der waals complexes: rare-gas dimers, alkaline-earth dimers, zinc dimer, and zinc-rare-gas dimers. *J. Phys. Chem. A* **110**, 5121–5129 (2006).

(17) Zhao, Y. & Truhlar, D. G. Density functional for spectroscopy: no long-range self-interaction error, good performance for rydberg and charge-transfer states, and better performance on average than b3lyp for ground states. *J. Phys. Chem. A* **110**, 13126–13130 (2006).

(18) Head-Gordon, M., Pople, J. A. & Frisch, M. J. MP2 energy evaluation by direct methods. *Chem. Phys. Lett.* **153**, 503–506 (1988).

(19) Cížek, J., Advances in Chemical Physics. (Wiley Interscience, New York, 1969).

(20) Pople, J. A., Head-Gordon, M., & Raghavachari, K. Quadratic configuration interaction. A general technique for determining electron correlation energies. *J. Chem. Phys.* **87**, 5968–5975 (1987).
